# Size of company of the longest‐held job and mortality in older Japanese adults: A 6‐year follow‐up study from the Japan Gerontological Evaluation Study

**DOI:** 10.1002/1348-9585.12115

**Published:** 2020-02-25

**Authors:** Satoru Kanamori, Taishi Tsuji, Tomoko Takamiya, Hiroyuki Kikuchi, Shigeru Inoue, Daisuke Takagi, Yuko Kai, Mitsuya Yamakita, Yoshito Kameda, Katsunori Kondo

**Affiliations:** ^1^ School of Nursing Tokyo Women's Medical University Tokyo Japan; ^2^ Department of Preventive Medicine and Public Health Tokyo Medical University Tokyo Japan; ^3^ Department of Social Preventive Medical Sciences Center for Preventive Medical Sciences Chiba University Chiba Japan; ^4^ Department of Health and Social Behavior Graduate School of Medicine The University of Tokyo Tokyo Japan; ^5^ Physical Fitness Research Institute Meiji Yasuda Life Foundation of Health and Welfare Tokyo Japan; ^6^ College of Liberal Arts and Sciences Kitasato University Kanagawa Japan; ^7^ Center for Well‐being and Society Nihon Fukushi University Aichi Japan; ^8^ Center for Gerontology and Social Science National Center for Geriatrics and Gerontology Aichi Japan

**Keywords:** health status disparities, Japan, mortality, occupational health

## Abstract

**Objectives:**

Very few longitudinal studies have investigated the question of whether differences in company size may give rise to health inequalities. The aim of this study was to examine the relationship between company size of the longest‐held job and mortality in older Japanese adults.

**Methods:**

This study used longitudinal data from the Japan Gerontological Evaluation Study. Surveys were sent to functionally independent individuals aged 65 or older who were randomly sampled from 13 municipalities in Japan. Respondents were followed for a maximum of 6.6 years. The Cox proportional hazards model was used to calculate mortality hazard ratios (HRs) for men and for women. Analysis was carried out on 35 418 participants (197 514 person‐years).

**Results:**

A total of 3935 deaths occurred during the 6‐year follow‐up period. Among men, in Model 1 that adjusted for age, educational attainment, type of longest‐held job, and municipalities, mortality HRs decreased significantly with increasing size of company (*P* for trend = .002). Compared to companies with 1‐9 employees, the mortality HR (0.78, 95% confidence interval: 0.68‐0.90) was significantly lower for companies with 10 000 or more employees. However, there were no significant differences among women (*P* for trend = .41).

**Conclusions:**

In men, mortality in old age may decrease with increasing size of company of the longest‐held job. To reduce health inequalities in old age due to differences in size of company, studies should be conducted to determine the underlying mechanisms and moderating factors and those findings should be reflected in labor policies and occupational health systems.

## 1 INTRODUCTION

After the Second World War, Japan became one of the healthiest countries in the world through its universal health insurance system and equal access to opportunities for education and medical care and has reduced health inequalities since.^1^ In recent years, however, health inequalities are trending upwards as the socioeconomic gap widens.^1^, ^2^ Size of company could be one factor associated with health inequalities under the scope of occupation.

In Japan, the Industrial Safety and Health Act requires health supervisors and occupational health physicians to be appointed to workplaces with 50 employees or more but not to workplaces with less than 50 employees.^3^ As a result, smaller workplaces have lower quality industrial health and safety activities.^4^, ^5^ They may also offer lower salaries on average.^6^ Considering life course,^7^ these differences due to company size may lead to health inequalities in the future. The average length of employment is particularly long among Japanese male workers compared to other workers in other countries,^8^ and determining whether differences in longest‐held job with company size are leading to health inequalities in Japan could provide useful information.

In the working generation, previous cross‐sectional studies have found a lower frequency of current smokers,^9^ daily drinkers,^9^ problem drinkers,^10^ health examination non‐participation,^11^ cancer screening non‐participation,^12^ and abnormalities in various health check‐ups items (eg, blood pressure and blood sugar),^9^ and lower scores for depressive symptoms^13^ among workers in large companies than workers in small‐ and medium‐sized companies. Nevertheless, few studies have examined whether such health disparities due to differences in size of company carry over into old age.

In a previous cohort study on older adults in Japan, men who had been working at workplaces with 49 or fewer employees as the longest‐held job were reported to have a higher risk of poor instrumental activities of daily living (IADL) in old age than men at workplaces with 50 or more employees.^14^ In that study, IADL was examined but mortality was not, leaving the question of whether size of company of the longest‐held job is associated with mortality risk in old age unanswered. In addition, workplace size was only dichotomized into 50 or more and less than 50 employees in the study mentioned above, and therefore the dose‐response relationship is unclear.

To test the hypothesis that mortality risk decreases with increasing company size, we examine the relationship between size of company of the longest‐held job and mortality risk in older adults in Japan.

## 2 MATERIALS AND METHODS

### 2.1 Study design and participants

This study was a population‐based prospective cohort study conducted in Japan. It was based on a sample from the Japan Gerontological Evaluation Study (JAGES) carried out in 13 municipalities ranging geographically from Hokkaido in northernmost Japan to the Kyushu region in southernmost Japan from August 2010 to January 2012. JAGES is a population‐based gerontological survey aimed at clarifying the social determinants of health.^15^ The survey was sent to 95 827 individuals aged 65 or older who were not certificated for needed long‐term care at baseline. Certification of needed long‐term care is based on evaluation of the need for long‐term care according to uniform criteria for all of Japan,^16^ and municipalities maintain records on who has been certified. Participants were selected at random sampling in each municipality. Among participants who responded to the baseline survey, those with invalid responses for ID number, age, and/or sex were excluded. Participants were followed for a maximum of 2416 days (6.6 years) and those with missing values for company size of the longest‐held job, or who responded “I don't know” or “I have never had a job” to the question about company size and/or type of longest‐held job were also excluded. Agriculture/forestry/fishery workers were additionally excluded as their longest‐held job was often self‐employment.^17^, ^18^

### 2.2 Measures

#### 2.2.1 Mortality outcome

We retrieved death records from 2010 to 2016 (maximum: 6.6 years) from the government database of public long‐term care insurance.

#### 2.2.2 Size of company of the longest‐held job

To determine the size of company of their longest‐held job, an indicator was developed based on the comprehensive Japanese General Social Surveys^19^ carried out in Japan. Participants were asked, “Of all of your jobs to date, about how many people worked in the entire company or organization where you were employed the longest?” Choices were 1‐9 employees, 10‐49 employees, 50‐499 employees, 500‐9999 employees, 10 000 employees or more, “I don't know,” and “I have never had a job.”

#### 2.2.3 Covariates

Based on previous studies,^14^, ^20^, ^21^, ^22^ age (65‐69, 70‐74, 75‐79, 80, or 85 years or more), educational attainment (less than 9, 10‐12 years, or more than 12 years), type of longest‐held job (white‐collar: professional/technical or administrative, pink‐collar: clerical or sales/service, blue‐collar: skilled/labor, or other^15^), and municipalities were used as covariates.

To investigate the contribution of behavioral factors to the relationship between size of company of the longest‐held job and mortality, daily walking time (less than 30, 30‐59, or 60 minutes or more), frequency of fruit and vegetable consumption (less than once a day, once a day, or twice a day or more), alcohol consumption status (current drinker, past drinker, or non‐drinker), smoking status (never a smoker, past smoker, or current smoker), and frequency of health checkups (within 1 year, more than 2 years ago, or never) were used as mediators.

To investigate the role of illness in the relationship between size of company and mortality, self‐reported medical condition for three major diseases (cancer, heart disease, and stroke) in old age were used as mediators.

To examine other income‐mediated pathways, annual equivalized income (less than 2 million yen per year = low, 2‐3.99 million yen per year = middle, 4 million yen or more per year = high) in old age was used as one mediator. Annual equivalized income was calculated by dividing gross household income by the square root of the number of household members.

### 2.3 Statistics analysis

The Cox proportional hazards model was used to calculate mortality hazard ratios (HRs) for men and for women. Respondents who were lost to follow‐up because they moved were excluded. In each model, size of company of 1‐9 employees was set as the referent category. In Model 1, we adjusted for age, educational attainment, type of longest‐held job, and municipalities. To investigate the contribution of behavioral factors in the relationship between size of company of the longest‐held job and mortality, in Model 2, we adjusted for all the factors in Model 1 as well as walking time, frequency of fruit and vegetable consumption, alcohol consumption status, smoking status, and frequency of health checkups in old age. In Model 3, we adjusted for all the factors in Model 1 as well as self‐reported medical condition for three major diseases in old age. In Model 4, we adjusted for all the factors in Model 1 as well as annual equivalized income in old age. As type of job is strongly associated with size of company,^23^ we conducted stratified analysis by type of longest‐held job.

Dummy variables were set for all variables. Based on a previous study, 24 a “missing” category was used in the analysis to account for missing responses. Test of linear trends in mortality rates were conducted using ordinary scaling across categories of size of company of the longest‐held job. The threshold for significance was *P* < .05. All statistical analyses were conducted using IBM SPSS version 21.0.

## 3 RESULTS

**Figure 1:**
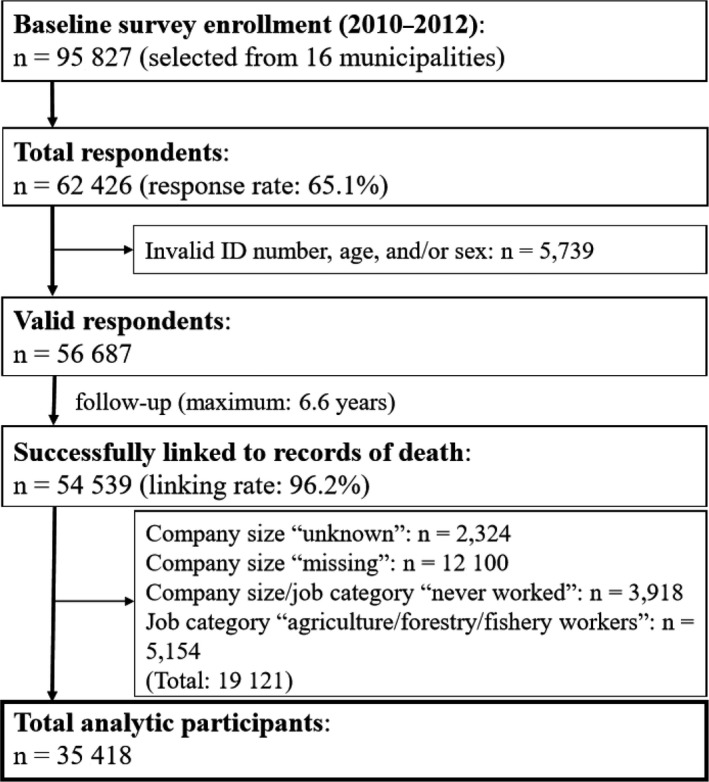
Flowchart of participants

Responses were received from 62 426 of the 95 827 individuals who were sent the questionnaire (response rate: 65.1%; Figure 1). Of these, 5739 were excluded for having an invalid response for ID number, age, and/or sex and 2148 because they could not be successfully linked to death records, leaving 54 539 valid respondents (25 146 men and 29 393 women). The job category of the longest‐held job was “agriculture/forestry/fishery workers” for 2608 men (2546 women) and never worked for 209 men (2764 women). After those who did not meet the required criteria (whose company size was unknown or missing, who never worked, or who were agriculture/forestry/fishery workers), the remainder was 35 418 participants who were used in the analysis. Participants were 19 260 men (54.4%) with a mean age of 73.3 ± 5.7 years and 16 158 women (45.6%) with a mean age of 72.9 ± 5.7 years.

The mean duration of follow‐up was 5.5 ± 1.3 years for men and 5.7 ± 1.0 years for women. During the follow‐up period, 2870 men (14.9%) and 1065 women (6.6%) died. The mortality rate per 1000 people was 27.2 for men and 11.6 for women.

Tables 1 and 2 show the characteristics of individuals by size of company of the longest‐held job for men and women. For men, the size of company of the longest‐held job was 1‐9 employees for 17.0% of men, 10‐49 employees for 21.9%, 50‐499 employees for 27.4%, 500‐9999 employees for 21.0%, and 10 000 or more employees for 12.6% (Table 1). For women, the size of company of the longest‐held job was 1‐9 employees for 25.9% of women, 10‐49 employees for 32.5%, 50‐499 employees for 27.8%, 500‐9999 employees for 9.9%, and 10 000 or more employees for 3.9% (Table 2).

**Table 1:** Individual characteristics of men according to size of company of the longest‐held job

	Size of company of the longest‐held job (number of employees)											
	1‐9		10‐49		50‐499		500‐9999		10 000+		Total	
	N	%	N	%	N	%	N	%	N	%	N	%
Total	3274	100.0	4213	100.0	5285	100.0	4053	100.0	2435	100.0	19 260	100.0
Age												
Mean ± SD	73.3 ± 5.6		73.4 ± 5.7		73.2 ± 6.7		73.0 ± 5.6		73.5 ± 5.9		73.3 ± 5.7	
Educational attainment												
Less than 6 y	64	2.0	64	1.5	56	1.1	27	0.7	10	0.4	221	1.1
6‐9 y	1750	53.5	1941	46.1	2074	39.2	1110	27.4	582	23.9	7457	38.7
10‐12 y	942	28.8	1178	28.0	1862	35.2	1503	37.1	1012	41.6	6497	33.7
13 y or more	472	14.4	961	22.8	1224	23.2	1354	33.4	806	33.1	4817	25.0
Type of longest‐held job												
White‐collar	1020	31.2	1484	35.2	1897	35.9	1751	43.2	1169	48.0	7321	38.0
Pink‐collar	910	27.8	924	21.9	1459	27.6	983	24.3	627	25.7	4903	25.5
Blue‐collar	636	19.4	926	22.0	1116	21.1	880	21.7	411	16.9	3969	20.6
Other	487	14.9	580	13.8	487	9.2	216	5.3	129	5.3	1899	9.9
Walking time												
Less than 30 min	1101	33.6	1353	32.1	1639	31.0	1107	27.3	591	24.3	5791	30.1
30‐59 min	931	28.4	1326	31.5	1832	34.7	1418	35.0	911	37.4	6418	33.3
60 min or longer	1077	32.9	1334	31.7	1610	30.5	1334	32.9	842	34.6	6197	32.2
Frequency of fruit and vegetable consumption												
Less than once a day	836	25.5	1126	26.7	1339	25.3	837	20.7	443	18.2	4581	23.8
Once a day	1108	33.8	1445	34.3	1832	34.7	1396	34.4	783	32.2	6564	34.1
Twice or more a day	1137	34.7	1427	33.9	1847	34.9	1595	39.4	1078	44.4	7084	36.8
Alcohol consumption status												
Current drinker	1704	52.0	2181	51.8	2862	54.2	2307	56.9	1444	59.3	10 498	54.5
Past drinker	202	6.2	267	6.3	348	6.6	247	6.1	128	5.3	1192	6.2
Non‐drinker	1174	35.9	1543	36.6	1813	34.3	1258	31.0	729	29.9	6517	33.8
Smoking status												
Has never smoked	790	24.1	939	22.3	1184	22.4	851	21.0	533	21.9	4297	22.3
Past smoker	1583	48.4	2070	49.1	2740	51.8	2263	55.8	1372	56.3	10 028	52.1
Current smoker	654	20.0	902	21.4	1023	19.4	689	17.0	382	15.7	3650	19.0
Health checkups												
Within 1 y	1678	51.3	2342	55.6	3183	60.2	2498	61.6	1524	62.6	11 225	58.3
2+ y ago	797	24.3	1049	24.9	1277	24.2	1059	26.1	654	26.9	4836	25.1
Never	650	19.9	669	15.9	658	12.5	412	10.2	202	8.3	2591	13.5
Self‐reported medical condition												
Cancer	192	5.9	243	5.8	338	6.4	258	6.4	162	6.7	1193	6.2
Heart disease	476	14.5	633	15.0	784	14.8	578	14.3	347	14.3	2818	14.6
Stroke	68	2.1	93	2.2	87	1.6	77	1.9	51	2.1	376	2.0
Annual equivalized income												
Low	1580	48.3	1920	45.6	2184	41.3	1302	32.1	647	26.6	7633	39.6
Middle	952	29.1	1352	32.1	2003	37.9	1967	48.5	1296	53.2	7570	39.3
High	329	10.0	430	10.2	522	9.9	470	11.6	320	13.1	2071	10.8

Note

Missing values for each factor have been omitted.

**Table 2:** Individual characteristics of women according to size of company of the longest‐held job

	Size of company of the longest‐held job (number of employees)											
	1‐9		10‐49		50‐499		500‐9999		10 000+		Total	
	N	%	N	%	N	%	N	%	N	%	N	%
Total	4177	100.0	5252	100.0	4492	100.0	1605	100.0	632	100.0	16 158	100.0
Age												
Mean ± SD	73.2 ± 5.8		73.2 ± 5.8		72.6 ± 5.5		72.1 ± 5.3		73.1 ± 5.6		72.9 ± 5.7	
Educational attainment												
Less than 6 y	112	2.7	125	2.4	83	1.8	20	1.2	0	0.0	340	2.1
6‐9 y	2019	48.3	2518	47.9	2032	45.2	599	37.3	175	27.7	7343	45.4
10‐12 y	1488	35.6	1763	33.6	1705	38.0	714	44.5	306	48.4	5976	37.0
13 y or more	481	11.5	752	14.3	587	13.1	255	15.9	141	22.3	2216	13.7
Type of longest‐held job												
White‐collar	516	12.4	732	13.9	627	14.0	188	11.7	80	12.7	2143	13.3
Pink‐collar	2109	50.5	2233	42.5	2028	45.1	910	56.7	418	66.1	7698	47.6
Blue‐collar	329	7.9	615	11.7	686	15.3	216	13.5	41	6.5	1887	11.7
Other	834	20.0	1111	21.2	762	17.0	180	11.2	50	7.9	2937	18.2
Walking time												
Less than 30 min	1413	33.8	1645	31.3	1311	29.2	444	27.7	163	25.8	4976	30.8
30‐59 min	1283	30.7	1812	34.5	1586	35.3	575	35.8	225	35.6	5481	33.9
60 min or longer	1240	29.7	1513	28.8	1349	30.0	496	30.9	201	31.8	4799	29.7
Frequency of fruit and vegetable consumption												
Less than once a day	635	15.2	878	16.7	649	14.4	183	11.1	54	8.5	2399	14.8
Once a day	1277	30.6	1540	29.3	1269	28.3	393	24.5	139	22.0	4618	28.6
Twice or more a day	2057	49.2	2585	49.2	2364	52.6	950	59.2	408	64.6	8364	51.8
Alcohol consumption status												
Current drinker	626	15.0	825	15.7	763	17.0	289	18.0	131	20.7	2634	16.3
Past drinker	37	0.9	58	1.1	50	1.1	20	1.2	12	1.9	177	1.1
Non‐drinker	3298	79.0	4091	77.9	3450	76.8	1207	75.2	456	72.2	12 502	77.4
Smoking status												
Has never smoked	3361	80.5	4249	80.9	3623	80.7	1326	82.6	522	82.6	13 081	81.0
Past smoker	239	5.7	283	5.4	247	5.5	88	5.5	40	6.3	897	5.6
Current smoker	149	3.6	195	3.7	194	4.3	49	3.1	21	3.3	608	3.8
Health checkups												
Within 1 y	2324	55.6	3104	59.1	2719	60.5	1003	62.5	417	66.0	9567	59.2
2+ y ago	912	21.8	1112	21.2	1000	22.3	352	21.9	138	21.8	3514	21.7
Never	780	18.7	787	15.0	588	13.1	175	10.9	57	9.0	2387	14.8
Self‐reported medical condition												
Cancer	131	3.1	157	3.0	122	2.7	43	2.7	27	4.3	480	3.0
Heart disease	375	9.0	460	8.8	385	8.6	140	8.7	62	9.8	1422	8.8
Stroke	30	0.7	38	0.7	18	0.4	8	0.5	3	0.5	97	0.6
Annual equivalized income												
Low	1794	42.9	2226	42.4	1868	41.6	568	35.4	145	22.9	6601	40.9
Middle	1159	27.7	1616	30.8	1489	33.1	659	41.1	322	50.9	5245	32.5
High	467	11.2	448	8.5	402	8.9	164	10.2	95	15.0	1576	9.8

Note

Missing values for each factor have been omitted.

Tables 3 and 4 show the mortality HRs for the size of company of the longest‐held job. Among men, in a trend test, mortality HR decreased significantly with increasing size of company in Model 1 that adjusted for age, educational attainment, type of longest‐held job, and municipalities (*P* = .002) (Table 3). In this model, only a company size of 10 000 employees or more had a significantly lower HR than a company size of 1‐9 employees. In Model 2 that also adjusted for behavioral factors in old age, the trend became marginal (*P* = .051). In addition, the HRs for company size of at least 50 employees all approached 1. In Model 3 that adjusted for self‐reported medical condition for three major diseases in old age in addition to the conditions in Model 1, the HRs did not differ significantly from those in Model 1. In Model 4 that adjusted for annual equivalized income in old age in addition to the conditions in Model 1, the HRs for company size of 500 employees or more all approached 1 but the changes in HR were smaller than Model 2. Among women, there were no significant associations in any of the models (Table 4).

**Table 3:** Mortality hazard ratios for the size of company of the longest‐held job among men

	N	Deaths	Person‐years	Model 1		Model 2		Model 3		Model 4	
				HR	95％CI	HR	95％CI	HR	95％CI	HR	95％CI
Total	19 260	2870	105 324								
1‐9	3274	544	17 807	ref		ref		ref		ref	
10‐49	4213	677	23 017	0.96	0.86‐1.08	0.96	0.86‐1.08	0.96	0.86‐1.07	0.96	0.86‐1.08
50‐499	5285	787	28 950	0.92	0.83‐1.03	0.94	0.84‐1.05	0.92	0.82‐1.02	0.93	0.83‐1.04
500‐9999	4053	558	22 210	0.93	0.82‐1.05	0.97	0.86‐1.09	0.92	0.82‐1.04	0.95	0.84‐1.08
10 000+	2435	304	13 341	0.78	0.68‐0.90	0.84	0.72‐0.97	0.77	0.67‐0.89	0.81	0.70‐0.93
*P* for trend				.002		.051		.001		.01	
White‐collar^a^	7321	983	40 139								
1‐9	1020	145	5613	ref		ref		ref		ref	
10‐49	1484	215	8147	0.99	0.80‐1.22	1.02	0.82‐1.26	0.98	0.79‐1.21	1.00	0.81‐1.24
50‐499	1897	260	10 390	0.92	0.75‐1.13	0.95	0.77‐1.17	0.91	0.74‐1.12	0.93	0.76‐1.15
500‐9999	1751	211	9638	0.92	0.74‐1.14	0.97	0.78‐1.21	0.93	0.75‐1.15	0.94	0.76‐1.18
10 000+	1169	152	6352	0.94	0.75‐1.19	1.01	0.80‐1.28	0.94	0.74‐1.18	0.98	0.77‐1.24
*P* for trend				.47		.88		.48		.66	
Pink‐collar^b^	4903	703	26 762								
1‐9	910	154	4900	ref		ref		ref		ref	
10‐49	924	138	5029	0.91	0.72‐1.14	0.88	0.70‐1.11	0.90	0.72‐1.14	0.90	0.72‐1.14
50‐499	1459	195	8047	0.86	0.70‐1.07	0.86	0.69‐1.07	0.86	0.69‐1.06	0.87	0.70‐1.09
500‐9999	983	141	5352	0.98	0.77‐1.24	0.97	0.77‐1.23	0.96	0.76‐1.22	1.02	0.80‐1.29
10 000+	627	75	3434	0.66	0.50‐0.87	0.67	0.51‐0.89	0.63	0.47‐0.83	0.69	0.52‐0.92
*P* for trend				.03		.051		.01		.09	
Blue‐collar^c^	3969	634	21 944								
1‐9	636	104	3519	ref		ref		ref		ref	
10‐49	926	174	5039	1.12	0.88‐1.43	1.12	0.88‐1.44	1.10	0.86‐1.40	1.12	0.88‐1.43
50‐499	1116	188	6172	1.00	0.78‐1.27	1.03	0.81‐1.31	0.98	0.77‐1.25	1.00	0.79‐1.27
500‐9999	880	131	4860	1.02	0.78‐1.33	1.09	0.84‐1.41	0.98	0.75‐1.27	1.02	0.79‐1.33
10 000+	411	37	2354	0.59	0.41‐0.86	0.65	0.44‐0.95	0.58	0.40‐0.85	0.60	0.41‐0.87
*P* for trend				.03		.14		.02		.03	
Other	1899	328	10 260								
1‐9	487	105	2573	ref		ref		ref		ref	
10‐49	580	89	3200	0.74	0.55‐0.99	0.70	0.52‐0.93	0.75	0.56‐1.001	0.75	0.56‐1.01
50‐499	487	79	2610	0.82	0.61‐1.11	0.84	0.62‐1.13	0.81	0.60‐1.15	0.86	0.64‐1.15
500‐9999	216	37	1176	0.75	0.52‐1.10	0.75	0.51‐1.11	0.79	0.54‐1.15	0.81	0.55‐1.19
10 000+	129	18	701	0.60	0.36‐1.00	0.61	0.36‐1.02	0.56	0.33‐0.94	0.65	0.39‐1.10
*P* for trend				.047		.07		.04		.13	

Note

Model 1 adjusts for age, educational attainment, type of longest‐held job (only in analysis of all participants), and municipalities.

Model 2 adjusts for age, educational attainment, type of longest‐held job (only in analysis of all participants), municipalities, and behavioral factors (walking time, frequency of fruit and vegetable consumption, alcohol consumption status, smoking status, and health checkups).

Model 3 adjusts for age, educational attainment, type of longest‐held job (only in analysis of all participants), municipalities, and self‐reported medical condition for three major diseases (cancer, heart disease, and stroke) in old age.

Model 4 adjusts for age, educational attainment, type of longest‐held job (only in analysis of all participants), municipalities, and annual equivalized income in old age.

Abbreviation: HR, Hazards ratio.

Missing values for the type of longest‐held job have been omitted.

a White‐collar: professional/technical and administrative.

b Pink‐collar: clerical and sales/service.

c Blue‐collar: skilled/labor.

**Table 4:** Mortality hazard ratios for the size of company of the longest‐held job among women

	N	Deaths	Person‐years	Model 1		Model 2		Model 3		Model 4	
				HR	95％CI	HR	95％CI	HR	95％CI	HR	95％CI
Total	16 158	1065	92 190								
1‐9	4177	311	23 871	ref		ref		ref		ref	
10‐49	5252	350	30 108	0.89	0.76‐1.03	0.89	0.76‐1.03	0.89	0.76‐1.04	0.89	0.76‐1.04
50‐499	4492	284	25 531	0.95	0.80‐1.12	0.95	0.81‐1.12	0.95	0.81‐1.12	0.96	0.81‐1.13
500‐9999	1605	83	9149	0.87	0.68‐1.11	0.88	0.69‐1.13	0.87	0.68‐1.11	0.88	0.69‐1.13
10 000+	632	37	3531	0.94	0.66‐1.32	0.97	0.68‐1.36	0.89	0.63‐1.26	0.97	0.69‐1.37
*P* for trend				.41		.53		.35		.55	
White‐collar^a^	2143	125	12 246								
1‐9	516	34	2930	ref		ref		ref		ref	
10‐49	732	39	4212	0.84	0.52‐1.35	0.77	0.47‐1.24	0.82	0.51‐1.31	0.85	0.53‐1.37
50‐499	627	38	3556	1.04	0.65‐1.69	1.03	0.64‐1.67	1.01	0.63‐1.64	1.05	0.65‐1.71
500‐9999	188	7	1095	0.69	0.30‐1.57	0.70	0.31‐1.61	0.63	0.28‐1.45	0.70	0.31‐1.61
10 000+	80	7	453	1.34	0.58‐3.09	1.14	0.49‐2.66	1.37	0.59‐3.16	1.38	0.60‐3.18
*P* for trend				.82		.89		.91		.77	
Pink‐collar^b^	7698	439	43 731								
1‐9	2109	147	12 076	ref		ref		ref		ref	
10‐49	2233	127	12 744	0.89	0.70‐1.13	0.88	0.70‐1.12	0.94	0.74‐1.19	0.90	0.71‐1.15
50‐499	2028	109	11 432	0.95	0.74‐1.23	0.96	0.75‐1.24	0.97	0.76‐1.25	0.97	0.75‐1.25
500‐9999	910	38	5143	0.79	0.55‐1.13	0.81	0.56‐1.16	0.82	0.57‐1.18	0.81	0.56‐1.17
10 000+	418	18	2336	0.73	0.45‐1.21	0.73	0.44‐1.20	0.72	0.44‐1.18	0.78	0.47‐1.28
*P* for trend				.17		.21		.18		.26	
Blue‐collar^c^	1887	168	10 924								
1‐9	329	33	1897	ref		ref		ref		ref	
10‐49	615	58	3566	0.93	0.61‐1.44	0.96	0.62‐1.49	0.89	0.57‐1.38	0.92	0.60‐1.42
50‐499	686	61	3952	1.10	0.71‐1.70	1.11	0.71‐1.72	1.06	0.69‐1.65	1.09	0.71‐1.69
500‐9999	216	14	1275	0.94	0.50‐1.78	0.90	0.47‐1.70	0.92	0.49‐1.75	0.93	0.49‐1.76
10 000+	41	2	235	0.87	0.21‐3.68	1.00	0.24‐4.22	0.93	0.22‐3.91	0.88	0.21‐3.70
*P* for trend				.84		.89		.84		.85	
Other	2937	228	16 805								
1‐9	834	68	4783	ref		ref		ref		ref	
10‐49	1111	87	6380	0.89	0.64‐1.22	0.86	0.62‐1.18	0.89	0.65‐1.23	0.90	0.65‐1.24
50‐499	762	56	4346	0.94	0.65‐1.35	0.91	0.63‐1.31	0.95	0.66‐1.37	0.96	0.67‐1.37
500‐9999	180	11	1026	0.88	0.47‐1.68	0.86	0.45‐1.65	0.97	0.51‐1.86	0.88	0.46‐1.68
10 000+	50	6	270	1.59	0.69‐3.70	1.71	0.73‐3.99	1.44	0.61‐3.38	1.63	0.70‐3.80
*P* for trend				.89		.94		.82		.84	

Note

Model 1 adjusts for age, educational attainment, type of longest‐held job (only in analysis of all participants), and municipalities.

Model 2 adjusts for age, educational attainment, type of longest‐held job (only in analysis of all participants), municipalities, and behavioral factors (walking time, frequency of fruit and vegetable consumption, alcohol consumption status, smoking status, and health checkups).

Model 3 adjusts for age, educational attainment, type of longest‐held job (only in analysis of all participants), municipalities, and self‐reported medical condition for three major diseases (cancer, heart disease, and stroke) in old age.

Model 4 adjusts for age, educational attainment, type of longest‐held job (only in analysis of all participants), municipalities, and annual equivalized income in old age.

Missing values for the type of longest‐held job have been omitted.

Abbreviation: HR, Hazards ratio.

a White‐collar: professional/technical and administrative.

b Pink‐collar: clerical and sales/service.

c Blue‐collar: skilled/labor.

In stratified analysis by type of longest‐held job, no significant associations were observed in any of the models among male while‐collar workers (Table 3). In Model 1, the HR was significantly lower for a company size of 10 000 employees or more compared to a company size of 1‐9 employees for male pink‐collar, blue‐collar, and other workers. Among women, there were no associations between size of company and mortality (Table 4). Appendix S1 shows the mortality HR for the longest‐held job. Among men, the HR was significantly higher only for other workers compared to white‐collar workers. Among women, there were no associations between the longest‐held job and mortality.

## 4 DISCUSSION

In the present study, we investigated the relationship between size of company of the longest‐held job and mortality in older Japanese adults using a large cohort study. The results showed that, among men, mortality rate decreases as size of company of the longest‐held job increases. In addition, the mortality HR was lower in companies with 10 000 or more employees compared to companies with 1‐9 employees. No such associations were found for women.

Mortality is one comprehensive health outcome, and we discovered the novel finding that mortality is indeed associated with company size. Several previous studies have found lower risks of health outcomes (abnormalities in various health check‐ups items,^9^ depressive symptoms,^13^ and decline in IADL^14^) in larger companies compared to smaller companies. However, only a few longitudinal studies have examined this relationship. In addition, as we know, no previous research has examined the relationship with mortality. In the present study, our hypothesis that mortality risk would decrease with increasing size of company of the longest‐held job was supported only in men. A previous cross‐sectional study did not find consistently positive associations with increasing company size for psychological distress.^25^ This finding and our results suggest that there may be different associations with company size depending on cause‐specific mortality. In the present study, we were only able to examine all‐cause mortality, and further studies are needed to clarify this point.

Among women, on the other hand, there was no association between size of company and mortality. Similarly, in a previous cohort study, workplace size was associated with IADL decline in men but not in women.^14^ The results of our study were consistent with the results of that study. Many unmarried women began working full time after Second World War, but most eventually quit for marriage or to have and raise children, and they often chose part‐time work after their children were grown.^26^ This may be why the health‐related effects of working at a company are weaker compared to men.

Income, occupational hazards, lifestyle, occupational health services, job stress, social capital, social security/pension, and other factors have been identified as possible mechanisms for health inequalities in the scope of occupation.^20^ These factors may also contribute to differences in mortality with size of company of the longest‐held job. To examine the contribution of behavioral factors in old age to the relationship between size of company of the longest‐held job and mortality, we additionally adjusted for behavioral factors in old age in Model 2. Among men, of the health behaviors we examined, there tended to be a lower prevalence of unhealthy behaviors (walking less than 30 minutes per a day, eating fruits and vegetables less than once a week, current smoker, and not receiving health checkups) as the size of company increased. This trend has also been observed in previous studies.^9^, ^11^ Differences in the work environment (eg, industrial health and safety activities) in the past may be reflected in health behaviors that persist into old age. In Model 2, the significance disappeared and the HRs for men who had been working in a company with at least 50 employees as the longest‐held job were closer to 1 (50‐499: 0.94 [0.84‐1.05], 500‐9999: 0.97 [0.86‐1.09], and 10 000−: 0.84 [0.72‐0.97]) compared to the HRs in Model 1 (0.92 [0.83‐1.03], 0.93 [0.82‐1.05], and 0.78 [0.68‐0.90], respectively). This suggests that behavioral factors in old age may help shrink differences in mortality risk.

To examine the contribution of diseases and income in old age, we additionally adjusted for three major diseases in old age in Model 3, and annual equivalized income in old age in Model 4. Almost no changes in HRs were observed in Model 3, suggesting that prevalence of the diseases did not increase proportionately with size of company; the largest difference in prevalence of the three major diseases with difference in size of company was 0.9% for men (cancer: between 5.8% for 10‐49 employees and 6.7% for 10 000 or more employees). The percentage of participants receiving a health checkup within 1 year increased with increasing size of company, with a difference as high as 11.3% (1‐9 employees: 51.3%, 10 000 or more employees: 62.6%). The higher rate of having health checkups at larger companies may have resulted in earlier detection of the three major diseases. This may be why presence of the three diseases alone could not explain the association between size of company and mortality.

In the model that adjusted for annual equivalized income, we observed similar, although smaller, changes in HRs as the model that adjusted for behavioral factors. Income may be particularly relevant, as salary tends to decrease with decreasing company size.^6^ In the present study as well, the ratio of participants with a lower annual equivalized income increased with decreasing size of company. In addition, a systematic review indicated that lower income is associated with a higher all‐cause mortality rate.^27^ This could explain why income contributes to the relationship between size of company and mortality.

Examinations of the pathways for these three factors suggest that health behaviors and annual equivalized income in old age may play a role in the relationship between size of company and mortality. One possible reason why HRs were only significantly lower in companies with 10 000 or more compared to companies with 1‐9 employees may be that differences in health behaviors are the strongest of the three factors. Further research is needed to clearly verify the indirect effects mediating behavioral factors, diseases, and income.

In analysis stratified by type of longest‐held job, there were no significant differences in the relationship between size of company and mortality among male white‐collar workers. Previous studies have examined how health outcomes are related to either company size or different types of jobs separately. Previous cohort studies that examined the relationship between type of job and health outcomes in Japanese people did not find any associations with decline of IADL^14^ or all‐cause mortality^21^ in either men and women. To our knowledge, ours is the first study to combine the company size and different types of jobs in one analysis. The finding that mortality risk does not differ by size of company for male white‐collar workers is novel. White‐collar workers have more job control than blue‐collar workers, so a high level of job control may correlate with low mortality risk^28^ and may be one factor protecting white‐collar workers from the effects of differences in size of company. However, this possibility was not directly explored in the present study, and further examination is needed.

In a study on a group of companies that carried out roughly the same activities for occupational safety and employed roughly the same labor regulations, no consistent associations were found in the relationship between size of company and health check‐ups items (eg, blood pressure and alanine aminotransferase).^29^ While that study points to the importance of industrial health and safety activities, one challenge that has been recognized in Japan is the lack of industrial health and safety activities at small‐ and medium‐sized companies.^5^ The extent of differences in the impact on future health for small‐ and medium‐sized companies compared to large companies had not previously been sufficiently clarified. The results of our study suggest that differences in company size affect mortality in old age. In other words, company size is one factor causing health inequalities. In Japan, small‐ and medium‐sized companies make up 99.7% of all companies and 68.8% of all employees work at small‐ and medium‐sized companies.^30^ Company size may therefore have a huge impact on mortality. Reducing inequalities requires more than just focusing solely on the most disadvantaged individuals. Activities scaled to the level of disadvantage should be rolled out universally as a type of proportionate universalism.^31^ The findings from the present study are therefore important evidence showing the necessity of dedicated measures for small‐ and medium‐sized companies and proportionate universalism tailored to company size. To consider such measures, research is needed to determine the mechanisms and mediating factors resulting in health inequalities in old age due to differences in size of company.

This study has some strengths. To the best of our knowledge, it is the first to examine the association between size of company of the longest‐held job and mortality risk in older adults. Furthermore, we used a large population‐based longitudinal dataset ranging from Hokkaido in northernmost Japan to the Kyushu region in southernmost Japan. However, it has several limitations. First is that the response rate was 65.1%, raising the possibility that this data does not provide a full picture of our study population. In addition, about one in four valid respondents were removed from analysis because their response to the question on size of company of the longest‐held job was “unknown” or missing (part of the exclusion criteria). Compared to respondents who were included in the analysis (men: 54.4%, women: 45.6%), those excluded respondents had a higher percentage of women (men: 28.5%, women: 71.5%). Caution must therefore be used especially when generalizing these results to women. In addition, the proportion of companies with 1‐9 employees (21.0%) and companies with 10‐49 employees (26.7%) were higher than proportions found in the 2014 economic census for business activity (9.3% and 19.4%, respectively).^30^ Type of work, work environment, and other factors over the long term may have affected the results of this study, making them less applicable to groups with other social backgrounds (eg, current workers who are under age 65). As the second limitation, the association between size of company and mortality rate may have been underestimated as we focused only on functionally independent individuals aged 65 or older and did not include those who became certified for need of long‐term care or died before we conducted our research. The third limitation was that we were unable to clearly separate participants who worked for companies and those who did not because they were self‐employed or were public servants, for example. Although we excluded agriculture/forestry/fishery workers as they are often self‐employed, our analysis may still have included others who did not work at a company. The fourth limitation was that self‐reported questionnaires were used in this research. Responses about past employment at companies may be affected by recall bias. Respondents who had not worked for many years or who had changed jobs numerous times may be especially vulnerable to recall bias. The fifth limitation was that we were limited to the types of indicators we could use. As size of company ranged quite broadly in our study, our categories differed from those often used in existing statistical data and previous studies. In addition, we were unable to examine the effects of lifestyle habits and health status prior to starting work at the longest‐held job, length of employment at the longest‐held job, or employment outside of the company of the longest‐held job. For health behaviors, we were only able to use frequency as an indicator. Future studies should take these points into account as well.

In conclusion, among Japanese men, mortality rate in old age may decrease with increasing size of company of the longest‐held job. To reduce health inequalities due to differences in size of company, the mechanisms and mediating factors need to be determined and reflected in labor policies.

## DISCLOSURES

*Approval of the research protocol*: The Research Ethics Committee of the Nihon Fukushi University Ethics Committee (application number: 10‐05) reviewed and approved the aims and procedures of this study. *Informed consent*: Informed consent was obtained from all individual participants included in the study. *Registry and the registration no. of the study/trial*: N/A. *Animal studies*: N/A. *Conflict of interest*: Authors declare no conflict of interests for this article.

## AUTHOR CONTRIBUTIONS

SK conducted the analysis and wrote the manuscript in collaboration with T Tsuji, T Takamiya, HK, and SI, and DT wrote the first draft of the manuscript. YK, MY, YK, and KK provided the feedback and suggestions. All authors read the manuscript and approved to submission.

## ETHICAL APPROVAL

Ethical approval for the study was obtained from the Nihon Fukushi University Ethics Committee (application number: 10‐05). This study was performed in accordance with the principles of the Declaration of Helsinki. Informed consent was obtained from all participants.
