# Supplementary material for: Size of company of the longest‐held job and mortality in older Japanese adults: A 6‐year follow‐up study from the Japan Gerontological Evaluation Study
Source: J Occup Health. 2020 Feb 25;62(1):e12115. doi: 10.1002/1348-9585.12115 (PMC7176136; doi:10.1002/1348-9585.12115)
Supplement: Supplementary file 1 [file JOH2-62-e12115-s001.docx]

**APPENDICES**

Appendix S1. Mortality hazard ratios for the type of longest-held job

|  | N | Deaths | Person -years | Model 1 | |
| --- | --- | --- | --- | --- | --- |
|  |  |  |  | HR | 95％CI |
| **Men** | 18,092 | 2,648 | 99,105 |  |  |
| White-collar^a^ | 7,321 | 983 | 40,139 | ref |  |
| Pink-collar^b^ | 4,903 | 703 | 26,762 | 1.09 | 0.99-1.21 |
| Blue-collar^c^ | 3,969 | 634 | 21,944 | 1.04 | 0.94-1.16 |
| Other | 1,899 | 328 | 10,260 | 1.16 | 1.02-1.32 |
|  |  |  |  |  |  |
| **Women** | 14,665 | 960 | 83,706 |  |  |
| White-collar^a^ | 2,143 | 125 | 12,246 | ref |  |
| Pink-collar^b^ | 7,698 | 439 | 43,731 | 1.03 | 0.84-1.25 |
| Blue-collar^c^ | 1,887 | 168 | 10,924 | 1.22 | 0.96-1.56 |
| Other | 2,937 | 228 | 16,805 | 1.06 | 0.85-1.32 |

Model 1 adjusts for age, educational attainment, and municipality.

^a^ White-collar: professional/technical and administrative

^b^ Pink-collar: clerical and sales/service

^c^ Blue-collar: skilled/labor

Missing values for the type of longest-held job have been omitted.
